# Neural correlates of asymmetric switch costs during affective task switching

**DOI:** 10.3758/s13415-026-01436-y

**Published:** 2026-04-02

**Authors:** Leif E. Langsdorf, Sebastian Kübler, Maryam Sadeghi Talarposhti, Torsten Schubert

**Affiliations:** 1 Institute for Psychology, Martin Luther University Halle-Wittenberg, Halle, Germany; 2 Institute for Psychology, Humboldt University, Berlin, Germany

**Keywords:** Affective task switching, Proactive cognitive control, Electroencephalography, Posterior positivity

## Abstract

Task switching is widely used to investigate cognitive control, typically revealing switch costs—greater performance costs in switch than in repetition trials. Previous research reported affective asymmetric switch costs, with higher costs when switching towards an affective task compared with a switch towards a neutral task. This asymmetry can be explained by the inhibition of the affective task, which enables the performance of the neutral task, leading to increased costs for a switch *from* the neutral *toward* the affective task. In Experiment 1, we examined whether the affective task content interferes with the preparation of the switching process, as reflected in neural correlates of proactive control. In Experiment 2, we investigated whether the maintenance of task sets is modulated by affective task content indexed by mixing costs (repetition – single-task performance) at the behavioural and the neural levels. Participants completed a cued task-switching during electroencephalographic recording, and judged either the gender (neutral-task) or emotional expression (affective-task) of faces. Neurally, within the cue-stimulus interval, the switch-related posterior positivity was reduced in the affective compared with the neutral task, coinciding with increased switch costs for the affective task. This suggests an impaired task-set reconfiguration for the affective compared with the neutral task. For Experiment 2, we investigated whether mixing costs and the mixing-related centroparietal positivity are modulated by affective task content. Neurally, within the cue-stimulus interval, the mixing-related centroparietal positivity remained unchanged by affective task content, mirrored behaviourally by symmetrical mixing costs. These findings indicate that affective task content selectively interferes with the preparation of the switching process but not with goal-setting.

Humans demonstrate the remarkable ability to flexibly switch between multiple tasks. Over the past decades, numerous studies have investigated this ability and identified the underlying cognitive control processes and their neural correlates (Karayanidis & Jamadar, 2014; Kiesel et al., 2010; ). Although research on cognitive control has made significant progress in defining the processes required for flexibly switching between tasks, the role of affective processing in modulating cognitive control in task switching has been largely overlooked. Recent studies have begun to address this issue; yet further investigations are required on how affective task content modulates neural dynamics of cognitive control (Eckart et al., 2021, 2023; Johnson, 2009; Maydych et al., 2024;Reeck & Egner, 2015; Schuch et al., 2012; Zhou et al., 2024). Addressing this gap, in the present study, we applied electroencephalography (EEG), which allows for a fine-grained temporal resolution of the measurement, to investigate how the neural dynamics and the related markers of cognitive control are modulated when participants switch between affective and neutral tasks.

## Task switching

An often-used paradigm to investigate cognitive control mechanisms is the task-switching paradigm. In this paradigm, participants are asked to switch (changing task compared with the previous trial) or repeat (same task compared with the previous trial) between two sensory-motor reaction time (RT) tasks in quick succession. This leads to the robust finding of switch costs, i.e., larger RTs and/or error rates for switching than repeating the tasks relative to the preceding trial (Wylie & Allport, 2000). To account for the phenomenon of switch costs, two types of accounts have been proposed (Kiesel et al., 2010). Top-down accounts, on the one hand, assume that switch costs reflect additional reconfiguration processes that are required in switch trials but not in repetition trials. In particular, it is assumed that the switching process relies on the activation of a new task set, e.g., the activation of a task goal, implementing the task rules, and inhibiting the previous task set that is no longer required (Rogers & Monsell, 1995). Conversely, bottom-up accounts assume that switch costs are emerging rather passively due to interference from the previously implemented task set that dissipates over time. Here, interference is related to the remaining activation of the previous task set, which requires inhibition in the current trial, resulting in additional processing costs (Alport et al., 1994). By now, researchers agree that both types of accounts are required to explain task-switching performance (Meiran et al., 2000; Poljac & Yeung, 2014; Yeung & Monsell, 2003).

In addition to the cognitive processes underlying task switching, in recent years, research has also addressed their neural correlates. These studies often applied the cued task-switching methodology in which participants are presented with a cue before stimulus onset, which signals to participants the task they should perform next. During the preparatory interval, event-related potentials (ERP) components associated with proactive control can be measured. One such ERP component is a cue-locked P3b-like posterior positivity peaking between 400 and 700 ms after cue presentation in the switch compared with the repetition condition (Donchin, 1981; Goffaux et al., 2006; Karayanidis & Jamadar, 2014; Karayanidis et al., 2009; Kieffaber & Hetrick, 2005; Kok, 2001; Polich, 2007; Poljac & Yeung, 2014; Rushworth et al., 2002; Steinhauser & Steinhauser, 2019; Verleger et al., 2005; Wylie et al., 2009)*.* This *switch-related posterior positivity* is presumably linked to a context-updating mechanism that is activated during switch trials compared with repetition trials when the internal task representation does not match the current task requirements, as signaled by the cue. Researchers have, furthermore, proposed that the increased positivity in the switch condition, in contrast to the repetition condition, reflects the updating of the mental representation for task-set reconfiguration; additionally, it seems possible that it reflects the updating of the stimulus–response rules within working memory (Jamadar et al., 2010; Karayanidis & Jamadar, 2014; Monsell, 2017; Periáñez & Barceló, 2009; Rogers & Monsell, 1995).

Previous task-switching studies have further specified the relationship between the amplitude of the cue-locked switch-related posterior positivity and switch costs on RTs. Karayanidis et al. (2011) demonstrated that the amplitude of the cue-locked switch-related posterior positivity was increased during trials with smaller RT switch costs but decreased for trials with larger RT switch costs (Jost et al., 2008; Lavric et al., 2008; Poljac & Yeung, 2014). Accordingly, researchers have assumed that the amplitude of the cue-locked switch-related posterior positivity indicates how well the processes involved in switching between tasks are initiated before stimulus onset. Indeed, this interpretation is compatible with the assumption that task-set reconfiguration can be prepared proactively and be initiated in switch trials before stimulus presentation (Jamadar et al., 2010; Karayanidis & Jamadar, 2014; Karayanidis et al., 2009; Rogers & Monsell, 1995). While most research has focused on the cognitive and neural mechanisms underlying task switching and their behavioral and neural markers, an often-overlooked question is how affective task content can modulate these cognitive control mechanisms.

## Cognitive control in affective task-switching

There is initial evidence that affective task content can modulate cognitive control in task switching, reflected in both behavioral performance and neural correlates (Eckart et al., 2023; Maydych et al., 2024; Reeck & Egner, 2015). For example, Reeck and Egner (2015) applied functional magnetic resonance imaging (fMRI) while participants were asked to classify affectively expressive faces according to the gender as male/female (i.e., neutral task) or the emotional expression as fearful/happy (i.e., affective task). The authors reported increased switch costs when participants switched *from* the neutral task *toward* the affective task compared with a switch in the opposite direction, i.e., reflecting affective asymmetric switch costs (Alport et al., 1994; Meuter & Allport, 1999; Poljac & Yeung, 2014; Schuch et al., 2012; Yeung & Monsell, 2003). Furthermore, the fMRI results indicated decreased activation in a right-lateralized frontostriatal network for conditions that are associated with increased behavioral processing costs when switching toward the affective task. The observation of this inverse relationship between neural activation and behavioral processing costs seems to indicate that the amount of activation in the network reflects the success with which control is implemented over the task sets. For the particular case of switching toward the affective task, Reeck and Egner (2015) suggested that the activity in the network is related to the successful release of the affective task set from inhibition by the former processing of the neutral task.

This interpretation is based on the assumption that affective asymmetric switch costs are related to the inhibition of the affective task set (Eckart et al., 2023; Öhman et al., 2001; Piguet et al., 2013; Reeck & Egner, 2011). In more detail, because of the dominance of the emotional information, the affective task set starts with a higher activation level than the neutral task set in working memory. Because both task sets generate mutual interference with each other during task switching, the affective task set needs to be inhibited to enable proper performance of the neutral task set. As a consequence, this results in persisting gradual inhibition of the affective task set and to larger switch costs when switching towards the affective compared with the neutral task (Declerck & Philipp, 2015; Declerck et al., 2015; Philipp & Koch, 2009). Indeed, the increased switch costs for the affective task were accompanied by decreased activity in the right-lateralized frontal network that is involved in inhibitory control of the affective task content and by enhanced coupling to the amygdala in switch versus repetition trials (Reeck & Egner, 2015). Several studies have reported affective asymmetric switch costs in situations in which participants switched between the performance of an affective task and a neutral task (De Vries & Geurts, 2012; Eckart et al., 2021, 2023; Rademacher et al., 2023; Reeck & Egner, 2015; Schuch et al., 2012; Zhou et al., 2024). However, in contrast to the accumulating evidence on the behavioral level, the neural dynamics of affective task switching—and affective asymmetric switch costs in particular—remain underspecified, requiring neurocognitive investigations with high temporal resolution.

Electroencephalography (EEG), with its millisecond-level temporal precision, provides a suitable tool to address how cognitive control unfolds during affective task switching. It allows for a temporally more fine-grained examination of the processing dynamic involved in the emergence of the asymmetric switch cost during affecting task-set switching. In particular, ERP analyses allow distinguishing between cue- and stimulus-locked components, which are most appropriate for this purpose. While the former components, such as the switch-related posterior positivity, reflect proactive control processes for the upcoming stimulus, the latter components index processes related to the stimulus itself (Rushworth et al., 2002; Sakai & Passingham, 2006). Zhou et al. (2024) recently applied this approach to investigate how affective task content modulates the neural implementation of task sets in a clinical and a control sample. Participants judged either the gender or the emotional expression of faces and produced affective asymmetric switch costs. Neurally, switching was associated with an enhanced frontocentral N2 and a reduced P3—described as a switch-related negativity—which was more pronounced during switching towards the affective task in the clinical sample compared with the control sample (Karayanidis & Jamadar, 2014; Tarantino et al., 2016). These results suggest that affective task content can modulate later ERP components during stimulus processing associated with the implementation of task sets. Relatedly, Elchlepp et al. (2021) reported increased switch costs in an emotional expression classification task compared with a letter classification task, and this was accompanied by a modulation of stimulus-locked ERP components (see also Leleu et al., 2012).

While earlier findings provide a relatively clear picture of the stimulus-locked ERP components and their modulation by the affective task content, it remains an open question whether and how affective task content modulates proactive control processes. These are assumed to be involved in the preparatory activity to task processing, which can be investigated by focusing on the cue-locked switch-related posterior positivity. Although this neural parameter has been robustly linked to proactive control, encompassing such processes as anticipatory task-set reconfiguration, its sensitivity to affective task content has yet to be established (Jost et al., 2008; Karayanidis & Jamadar, 2014; Karayanidis et al., 2009; Kieffaber & Hetrick, 2005; Poljac & Yeung, 2014; Rushworth et al., 2002). As a result, it remains an open question whether and how affective task content modulates neural correlates of proactive control, as reflected in the switch-related posterior positivity.

## The current study

In the current study, we investigated whether and how affective task content modulates proactive control processes, as reflected in cue-locked ERP components. We applied EEG in a cued task-switching paradigm and focused primarily on cue-locked ERP components of proactive control, in both repetition and switch conditions for neutral and affective tasks. We utilized tasks and stimulus material from the NimStim Set of Facial Expressions,^1^ which have been shown to reliably result in affective asymmetric switch costs (Eckart et al., 2023; Reeck & Egner, 2015; Tottenham et al., 2009). In each trial, a cue indicated which task participants should prepare. After the preparatory interval, a face stimulus was presented, which participants discriminated either according to gender (i.e., neutral task) or emotional expression (i.e., affective task).

In Experiment 1, we examined whether affective task content modulates the switching process between tasks, specifically whether it interferes with the anticipatory task-set reconfiguration when participants switch towards the affective compared with the neutral task. This was assessed via the cue-locked switch-related posterior positivity. In Experiment 2, we sought to extend our findings from Experiment 1 to a condition with a shorter preparatory interval and additionally focused on whether affective task content interferes with a further proactive control process, the maintenance of task sets in working memory, as indexed by the mixing costs (i.e., repetition minus single task performance) on behavioral and neural paramteres (for details see Experiment 2). These costs are perceived by many researchers as reflecting the maintenance of task sets in working memory, on which we focus in Experiment 2 (Braver et al., 2003; Los, 1996). Overall, based on previous investigations, it remains an open question whether and how ERP components of proactive control (and consequently the underlying control processes) are modulated by the requirement to switch between affective and neutral tasks.

Next, we turn to the predictions for Experiment 1 concerning how the affective task content can modulate the neural dynamics of proactive control and switch costs. Considering the behavioral results, we predicted that the switch costs, i.e., the RT difference between task switches and repetitions, should increase for the affective task compared with the neutral task, which would indicate affective asymmetric switch costs (Eckart et al., 2023; Reeck & Egner, 2015; Schuch et al., 2012). Next, we consider how affective task content might modulate the switch-related posterior positivity. Based on the assumption that affective tasks, compared with neutral ones, require greater inhibition, it is conceivable that this leads to impaired anticipatory task-set reconfiguration when participants switch *from* the neutral *towards* the affective task. This impairment may be reflected in a reduced amplitude of the cue-locked switch-related posterior positivity between 400 and 700 ms at electrode Pz in the affective compared with the neutral task. This reasoning is based on previous reports indicating that a reduced amplitude of the cue-locked switch-related posterior positivity reflects a poorer initiation of the switching processes (Karayanidis et al., 2011; Kieffaber & Hetrick, 2005; Poljac & Yeung, 2014; Wu et al., 2015).

Alternatively, it is also conceivable that participants show larger switch costs when switching towards the affective compared with the neutral task (Eckart et al., 2023; Reeck & Egner, 2015). This is not accompanied by a modulation of the cue-locked switch-related posterior positivity. Even if affective compared with neutral tasks are strongly inhibited, this may not affect the anticipatory task-set reconfiguration as reflected by this ERP component. Instead, the increased inhibition might primarily modulate stimulus-related processes, such as the implementation of task sets (Elchlepp et al., 2021; Zhou et al., 2024). This would be expected to result in a modulation of stimulus-locked ERP components when participants switch towards the affective task compared with the neutral task. Consistent with this assumption, previous studies have shown an increased switch-related negativity in the affective compared with the neutral task, as indexed by the stimulus-locked N2 and P3 components (Elchlepp et al., 2021; Zhou et al., 2024). In this case, one might not expect that switching between tasks is necessarily reflected in different amplitudes of the cue-locked switch-related posterior positivity between 400 and 700 ms at Pz but on the stimulus-locked ERP components.

Finally, it is conceivable that the affective task content modulates proactive control, and in addition, stimulus-related processes, as indexed by different ERP components. This could be reflected by a combination of the previously described outcomes, i.e., a reduction of the cue-locked switch-related posterior positivity in the affective compared with the neutral task, as well as an increased stimulus-locked switch-related negativity, as indexed by the N2 and P3 components, in the affective compared with the neutral task.

## Experiment 1

In Experiment 1, we investigated the neural dynamics of affective task switching to elucidate whether neural correlates of the affective asymmetric switch costs emerge during the preparatory interval. This would indicate an impaired anticipatory task-set reconfiguration in the affective compared with the neutral task and could suggest that inhibition diminished neural correlates of proactive control. To that end, we measured the cue-locked switch-related positivity over parietal sites to investigate neural preparatory processes before stimulus onset, while participants repeated and switched between a neutral and an affective face classification task. We were especially interested in measuring the neural dynamics of the switching processes and to elucidate whether differences emerge in the switch-related posterior positivity when switching from the gender classification towards the emotion-expression task and vice versa.

In addition to the main question of how the affective task content modulates proactive control, we investigated whether the affective task content also modulates stimulus-related processes, for which we focused on the switch-related negativity in the N2 and P3 windows. This allowed us to conduct a thorough investigation of how affective task content modulates the neural dynamics of the switching process.

### Methods

#### Participants

Twenty-four healthy participants (20 females; mean (m) age = 26 years) took part in the experiment. The sample size for both experiments was based on an a priori power analysis using the G*Power software (Faul et al., 2007). Based on the study by Johnson (2009), we estimated an effect size of η_G_^2^ = .103 (*f* = .33) for an interaction effect between the factors task and trial type on RTs. This interaction would indicate the occurrence of affective asymmetric switch costs for affective and neutral tasks. The analysis yielded a necessary sample size of *N* = 24 (with the other parameters determined as follows: α err prob = .05; Power (1 − β err prob) = .95). We based our a priori power analysis on RTs, rather than ERP components, as previous EEG studies had not reported the effect size for the interaction effect between the factors task and trial type on the components (Leleu et al., 2012; Zhou et al., 2024). To further increase statistical power, we invited participants to a training session before the EEG measurement (see *Method*) and included more than twice as many trials compared with the previous EEG investigations. Given these methodological changes, we assumed that our estimated sample size was sufficiently large to reliably detect an interaction effect between the factors task and trial type on the ERP components. This assumption was further supported by the results of Bayesian analyses,^2^ which indicated robust evidence for the presence of an interaction effect between the factors task and trial type in the neurocognitive data, which replicated across Experiments 1 and 2 (see *Results*) (Boudewyn et al., 2018). The experimental protocol conformed to the Declaration of Helsinki, and written informed consent was obtained from each participant before the start of the experiment. All participants were right-handed, German native speakers, and had normal or corrected-to-normal vision. Furthermore, participants could choose between 30 euros and course credit as payment. After the experiment, participants were debriefed.

#### Task and procedure

Participants sat in an electrically shielded experimental chamber in front of a 24-inch LG 24Bk55 computer screen with a resolution of 1920 × 1200 pixels and a refresh rate of 60 Hz, located at eye level with a distance of approximately 80 cm. Stimulus material comprised eight pictures of male and female faces showing a fearful or happy emotional expression (Tottenham et al., 2009). We choose the following pictures from the NimStim database: 01F_FE, 01F_HA, 02F_FE, 02F_HA, 06F_FE, 06F_HA, 09F_FE, 09F_HA, 24M_FE, 24_M_HA, 28M_FE, 28M_HA, 32M_FE, 32M_HA, 37M_FE, 37M_HA. We oval-cropped the stimulus material to standardize the size and shape of the facial stimuli with a diameter of 2.08° × 1.36° and applied a gray filter. All stimuli were presented on a black background. Participants were asked to categorize emotionally expressive faces either according to gender (male/female) or emotional expression (happy/fearful). To do so, participants used their left middle and index fingers to press the keys “D and F” and their right index and middle fingers to press the keys “J and K” on a QWERTZ keyboard to respond to the face stimuli. Thus, tasks were mapped to different hands. The experiment was run in PsychoPy v2022.1.2 (Peirce et al., 2019).

**Fig. 1:**
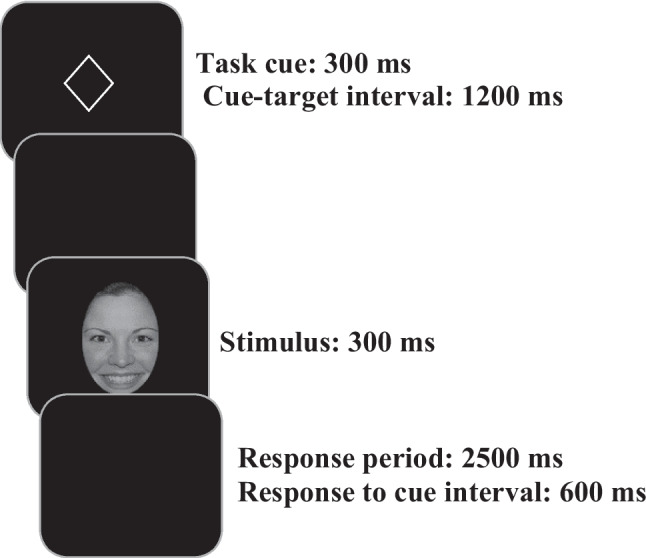
Trial structure for Experiment 1. A task cue is presented for 300 ms, signaling which task to prepare next, separated by a cue-target interval (CTI) of 1,200 ms, followed by a face stimulus for 300 ms. The maximal response period was 2,500 ms. The response-cue-interval was 600 ms. For Experiment 2, the CTI was reduced to 700 ms, whereas all other temporal parameters were identical to Experiment 1

Each trial started with the presentation of a cue for 300 ms, which was either a square or a rhombus with a visual angle of .82° × .82° (Fig. 1). The cue signaled to the participants which task to prepare. The cue was followed by a blank interval of 900 ms, summing up to a cue-target interval (CTI) of 1,200 ms. This CTI was chosen following previous studies that investigated ERPs of proactive control in task switching (Goffaux et al., 2006; Karayanidis et al., 2009; Mansfield et al., 2012; Rushworth et al., 2002; Steinhauser & Steinhauser, 2019). The face stimulus was presented for 300 ms. The response was followed by a blank screen for 600 ms (i.e., response-cue interval). The maximal response window was 2,500 ms. The cue-task as well as the response-task mapping was counterbalanced across participants, resulting in four experimental conditions to which participants were randomly assigned.

The study was conducted in two sessions separated by a maximum of 2 days in line with other studies on neural correlates of tasks witching (Karayanidis et al., 2009; Steinhauser & Steinhauser, 2019). In the first session, participants familiarized themselves with the stimulus set of 16 face stimuli. Subsequently, they performed three pure blocks, i.e., blocks consisting of only one task of 32 trials for the neutral task as well as the affective task. After the single-task blocks, participants performed eight mixed blocks of 65 trials, switching and repeating between the tasks, which resulted in 520 trials of task-switching practice, while after every 65 trials, a self-paced break was inserted. The experimental blocks of 65 trials were based on 16 face stimuli, which were each presented four times, resulting in 64 trials. To classify 64 trials as 32 repetition and 32 switch trials, one trial was added as the first trial, resulting in 65 trials, which was subsequently excluded from further data analysis. During the first session, participants received the feedback “Falsch” (German for wrong) for 500 ms if their response was erroneous.

In the second session, we measured the EEG. Before the start of the main part of the experiment, participants again familiarized themselves with the face stimuli. Subsequently, they performed one pure block of 32 trials each for the neutral and the affective tasks. Then, the main part of the experimental session started, in which they performed 13 mixed blocks of 65 trials, resulting in 845 trials, while in every 65 trials, a break occurred. After each block, participants received feedback on their mean RT and error rates for performance monitoring.

### Data acquisition

EEG was recorded by using an actiCHamp Plus system (actiCHamp Plus, Brain Products GmbH, Gilching, Germany). Thirty-two Ag/AgCl electrodes were placed, according to the international 10–20 system (Jasper, 1958) at the following sites: Fp1, Fp2, Fz, F3, F4, F7, F8, FC1, FC2, FC5, FC6, FT9, FT10, C3, C4, Cz, T7, T8, CP1, CP2, CP5, CP6, TP9, TP10, P3, P4, P7, P8, Pz, O1, Oz, and O2. The ground electrode was positioned between Fp1 and Fp2. TP10 served as the online reference, all electrodes were offline re-referenced to the average of TP9 and TP10, placed on the left and right earlobes. The horizontal electrooculogram (hEOG) was recorded from electrodes placed at the outer canthi of both eyes. Electrode impedance was kept below 10 kΩ. EEG and hEOG signals were recorded continuously at 500 Hz.

### Data analysis

#### Behavioral data

Mean RTs and error rates were separately analyzed using an analysis of variance (ANOVA) with the within-subject factors task (affective, neutral) and trial type (repetition, switch). A significance threshold of 5% was used for all analyses. For the RT analyses, trials with an erroneous response (*m* = 9.6%) and outliers that deviated more than ± 2.5 SD from the mean RTs for each participant and factor combination (*m* = 2.9*%*), as well as the first trial of each block, were excluded from the data set. All behavioral analyses and visualizations were conducted in R (R Core Team, 2021; Wickham, 2011; Wickham et al., 2019). To resolve relevant interaction effects, we applied paired two-sided Bonferroni-corrected *t*-tests.

We furthermore applied Bayesian ANOVAS and *t*-tests to further validate the results (Love et al., 2019; Wagenmakers et al., 2018). As our primary interest was the modulation of cognitive control by affective task content (i.e., interaction effect between the factors task and trial type), we focused on the relative evidence for the relevant model containing both main effects *and* the interaction term compared with the simpler model only containing both main effects. We divided the Bayes factor (*BF₁₀)* of the relevant model by that of the simpler model. If the outcome of this comparison yields a *BF₁₀* > 1, this indicates evidence for the inclusion of the interaction term, whereas a *BF₁₀* < 1 favors the simpler model. For relevant interaction effects, we used Bayesian paired t-tests, for which a *BF₁₀* > 1 supports the assumption that both measurements differ given the observed data, while a *BF₁₀* < 1 supports the assumption that measurements are equal given the observed data.

#### Electrophysiological data

All preprocessing steps were performed in BrainVision Analyzer Version 2.2, applying a customized script, while grand-averaged ERPs were exported to R for further statistical analyses and visualizations (BrainVision Analyzer, Version 2.2, Brain Products GmbH, Gilching, Germany). First, all data sets were visually inspected. We applied to the continuous EEG data an infinite impulse response (IIR) Butterworth Filter for frequencies below 0.5 Hz and above 30 Hz (12 dB/octave roll-off). The continuous data were epoched for correct response trials, for the cue-locked ERP components, from − 200 ms to 1,200 ms around cue onset, with a baseline correction applied to the 200 ms before cue onset. To capture stimulus-locked ERP components, the epoche length was extended by an additional 800 ms.

Epochs that contained a maximum amplitude exceeding ± 70 µV were excluded from further preprocessing steps. Maximally allowed differences in voltage steps were 200 µV within a 200-ms interval. Channels with low activity were identified by detecting segments in which the amplitude remained below 0.5 µV for at least 200 ms. If any of the above-listed criteria were met, the entire epoch was excluded from further preprocessing. To further correct eye movements and muscle artifacts, we visually checked the selection of the algorithms and applied an infomax-based independent component analysis (Bell & Sejnowski, 1995). This resulted in the exclusion of components with a time course and topography that are typical for those types of artifacts. We excluded participants for whom more than 40% of trials were rejected; based on this criterion, we excluded the data sets of two participants from the present data set. After preprocessing, participants contributed to the affective repetition condition on average 157 trials (minimum = 149, maximum = 192); for the affective switch condition, on average, 152 trials (minimum = 139, maximum = 188); for the neutral repetition condition, on average, 159 trials (minimum = 141, maximum = 199); and for the neutral switch condition, on average, 163 trials (minimum = 146, maximum = 204).

In line with previous task-switching investigations, we quantified the amplitude of the cue-locked switch-related posterior positivity at Pz within the CTI from 400 and 700 ms after cue presentation (Karayanidis & Jamadar, 2014; Karayanidis et al., 2009; Steinhauser et al., 2021). For that purpose, we computed the mean amplitude of this ERP component for each factor combination and utilized the mean amplitude as the dependent variable in an ANOVA with the factors task (neutral, affective) and trial type (repetition, switch), for which we applied a significance threshold of 5%.

To quantify the stimulus-locked switch-related negativity, we focused on the amplitudes of the N2 and P3 components at Cz (Gajewski & Falkenstein, 2011; Gajewski et al., 2010; Karayanidis & Jamadar, 2014). To determine the interval for statistical analysis, we applied the collapsed localizers approach and defined a 100-ms interval around the grand-averaged N2 peak and a 150-ms interval around the grand-averaged P3 peak to account for the broader distribution of the latter ERP component (Luck, 2014). We computed the mean amplitude of both ERP components for each factor combination and utilized them as the dependent variables in ANOVAs with the factors task (neutral, affective) and trial type (switch, repetition). We applied a significance threshold of 5%. We additionally, applied a Bayesian analysis of the ERP-related data to further validate the neurocognitive results.

### Results

#### Behavioral data

For RT data, we found a significant main effect of the factor task, *F*(1, 21) = 19.65, *p* < .001, η_G_^2^ = .10. Participants responded faster to the neutral task (*m* = 562 ms) compared with the affective task (*m* = 620 ms). We also obtained a significant main effect of the factor trial type, *F*(1, 21) = 7.96, *p* < .001, η_G_^2^ = .11, which indicates switch costs with shorter RTs in the repetition (*m* = 559 ms) compared with the switch (*m* = 623 ms) condition (Wylie & Allport, 2000). The repetition and switch RTs per task also differed significantly, *t*(21) = 3.40, *p* < .003, *d* = 0.72, *BF*_*10*_ = 14.7, and *t*(21) = 4.98, *p* < .001, *d* = 1.10, *BF*_*10*_ = 412.19, respectively.

**Fig. 2:**
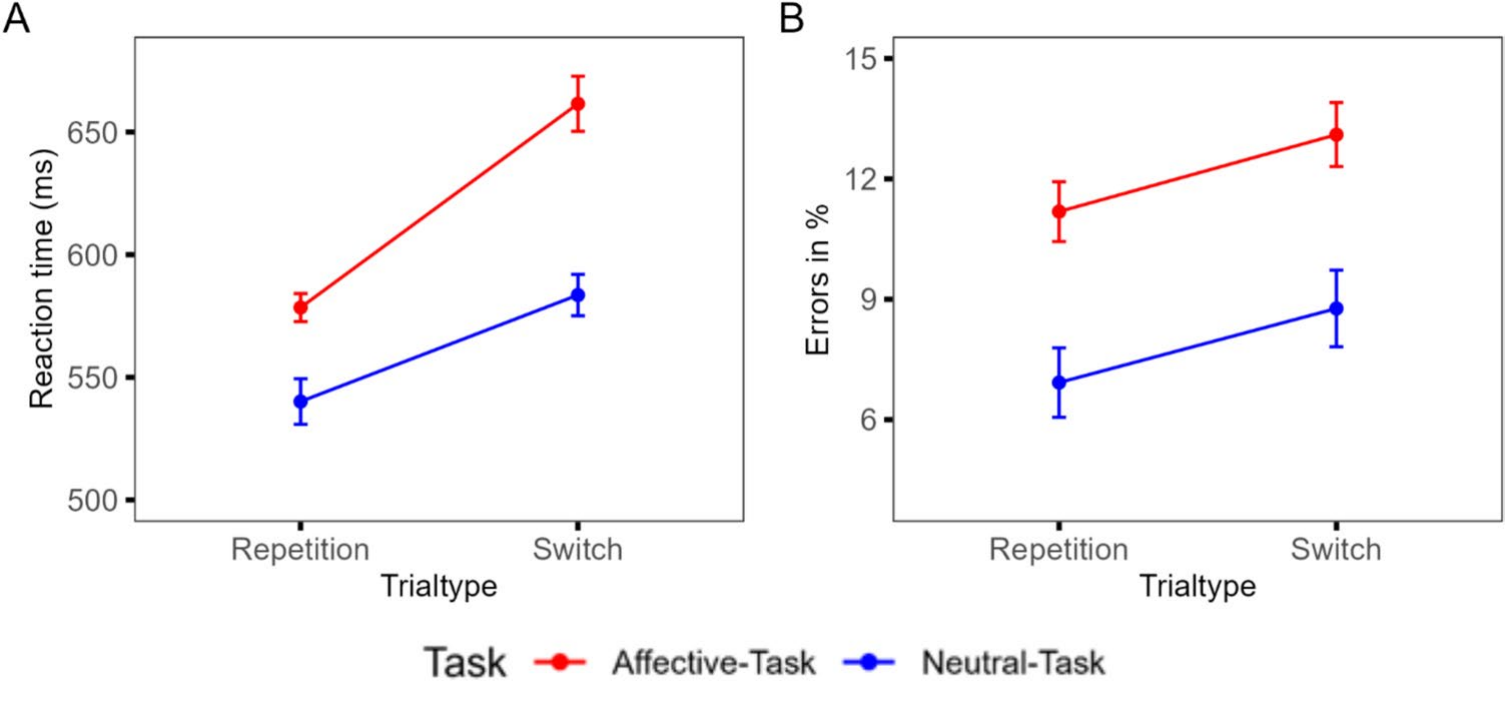
**A**) Mean reaction times (RTs) as a function of task and trial type. **B**) Error rates as a function of task and trial type. Error bars represent the standard error of the mean. The results indicate that the switch costs on RTs increase when participants switch from the neutral to the affective task compared with the opposite switch direction

More relevantly, for the issue of the affective asymmetric switch costs, the interaction effect between the factors task and trial type reached significance, *F*(1, 21) = 26.6, *p* < .001, η_G_^2^ = .01, *BF*_*10*_ = 188.78 (Fig. 2A). In particular, we obtained significant switch costs on the RTs for both tasks, i.e., for the affective task, *t*(21) =  − 10.22, *p* < .001, *d* =  − 2.2, *BF*_*10*_ = 8.42 × 10^6^, and for the neutral task, *t*(21) =  − 5.39, *p* < .001, *d* = 1.2, *BF*_*10*_ = 997.92, respectively. However, most importantly, we obtained larger switch costs for the affective task (*m* = 83 ms) compared with the neutral task (*m* = 43 ms), *t*(21) = 5.16, *p* < .001, *d* = 1.1, *BF*_*10*_ = 605.55. Hence, the switch costs increased when participants switched from the neutral task towards the affective task (Eckart et al., 2023; Reeck & Egner, 2015; Schuch et al., 2012).

We also analyzed the error rates and obtained a significant task effect, *F*(1, 21) = 27.46, *p* < .001, η_G_^2^ = .1: Participants made more errors executing the affective task (*m* = 12.1%) compared with the neutral task (*m* = 7.8%). Neither the main effect of trial type, *F*(1, 21) = 3.12, *p* = .092, η_G_^2^ = .01, nor the interaction effect between the factors task and trial type reached significance, *F*(1, 21) < 1.00, *p* = .952, η_G_^2^ = 4 × 10^–6^, *BF*_*10*_ = 0.32 (Fig. 2B).

### Event-related brain potentials

#### Cue-locked event-related potentials

**Fig. 3:**
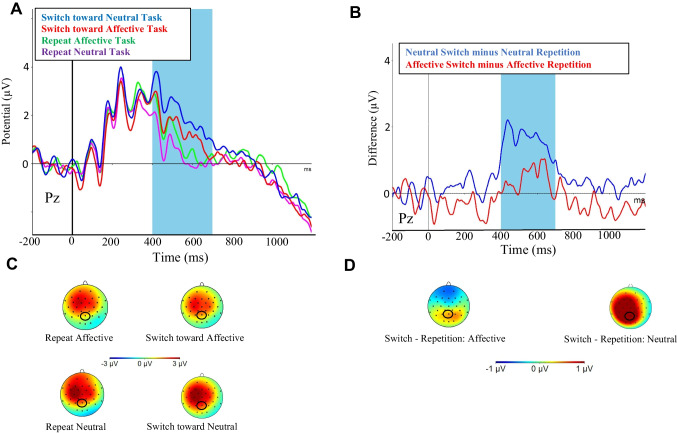
Cue-locked event-related potentials of correct trials at Pz during the cue-target interval, focusing on the switch-related posterior positivity. **A**) All four experimental conditions: the blue area marks the time interval used for statistical testing based on Steinhauser and Steinhauser (2019). **B**) The difference waves of the neutral switch minus neutral repetition (blue) and the affective switch minus affective repetition (red) condition. The topographical plot (**C**) is based on the four experimental conditions and (**D**) is based on the switch-repetition difference for the affective task and the neutral task, respectively. Both topographical plots focus on the time interval used for statistical testing (i.e., 400 ms and 700 ms after cue onset). The difference wave for the neutral compared with the affective task showed an increased positivity, which reflect a neural correlate of the affective asymmetric switch costs before stimulus onset

We obtained a typical P3b-like posterior positivity, commonly reported during task switching (Fig. 3A). This observation is further confirmed by the topographies showing a posterior spatial distribution of the component (Fig. 3C and D) (Karayanidis & Jamadar, 2014; Karayanidis et al., 2009; Steinhauser & Steinhauser, 2019). Mirroring the results on the behavioral level, we obtained a main effect of trial type on the amplitude measured at Pz, *F*(1, 21) = 19.81, *p* < .001, η_G_^2^ = .08. The posterior positivity was increased in switch (*m* = 1.1 µV) compared with repetition trials (*m* = 0.4 µV), which reflects a switch-related posterior positivity. Furthermore, we obtained significant differences per task on the amplitude of the posterior positivity in the repetition and the switch conditions, *t*(21) = 2.13,* p* < .045, *d* = 0.45, *BF*_10_ = 1.45, and *t*(21) =  − 2.80, *p* < .011, *d* =  − 0.59, *BF*_10_ = 4.69, respectively. In contrast, the effect of the factor task did not reach significance, *F*(1, 21) = .33, *p* = .572, η_G_^2^ = 7 × 10^–4^.

The amplitude of the switch-related posterior positivity was further modulated by affective task content, as reflected by an interaction effect between the factors task and trial type, *F*(1, 21) = 12.32, *p* < .002, η_G_^2^ = .03, *BF*_*10*_ = 87.32. In particular, we obtained significant switch costs on the amplitude of the posterior positivity in both tasks, for the affective task, *t*(21) =  − 2.12, *p* < .046, *d* =  − 0.45, *BF*_10_ = 1.44, and for the neutral task, *t*(21) =  − 4.69, *p* < .001, *d* =  − 1.00, *BF*_10_ = 225.96. Most importantly, difference waves—calculated by subtracting repetition trial amplitudes from switch trial amplitudes for each task—confirmed that the amplitude of the switch-related posterior positivity was increased in the neutral task (*m* = 1.6 µV) compared with the affective task (*m* = 0.5 µV), *t*(21) = 2.98, *p* < .002, *d* = 1.40, *BF*_10_ = 18.93 (Fig. 3B). This is consistent with the assumption that task-set reconfiguration was impeded due to the presumed persisting inhibition of the affective rather than the neutral task.

#### Stimulus-locked event-related potentials

**Fig. 4:**
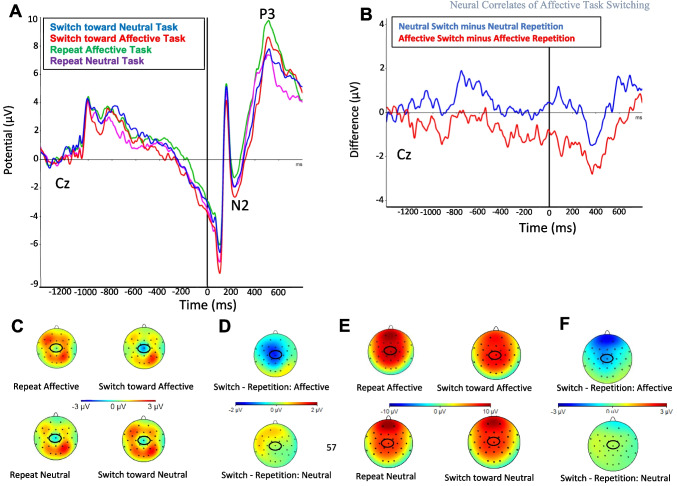
Stimulus-locked event-related potentials of correct trials at Cz, focusing on the switch-related negativity. **A**) All four experimental conditions, which depict the N2 and P3 components, respectively. **B**) The difference waves of the neutral switch minus neutral repetition (blue) and the affective switch minus affective repetition (red) conditions. The topographical plots (**C**) and (**D**) focus on the N2 component (i.e., 100-ms interval centered on the N2 peak), the former depicting the four experimental conditions and the latter depicting the switch-repetition difference for the affective task and the neutral task, respectively. The topographical plots (**E**) and (**F**) focus on the P3 component (i.e., 150-ms interval centered on the P3 peak), the former depicting the four experimental conditions and the latter depicting the switch-repetition difference for the affective task and the neutral task, respectively. The difference wave for the neutral compared with the affective task showed a decreased negativity in the N2 and an increased positivity in the P3 interval, reflecting a neural correlate of the affective asymmetric switch costs after stimulus onset

For the stimulus-locked ERP components, we obtained the typically reported frontocentrally distributed negativity for switch compared with repetition trials, more pronounced for the switch-repetition contrast in the affective task compared with the neutral task (Fig. 4A, C, and D) (Karayanidis & Jamadar, 2014; Tarantino et al., 2016). For the N2 component, we measured a negative peak at Cz 226 ms after stimulus presentation, which was used to define the center of the analysis interval (± 50 ms). Within the interval, the amplitude of the N2 component was modulated by the factor trial type, *F*(1, 21) = 5.28, *p* < .032, η_G_^2^ = .01, which reflects a more negative amplitude in switch (*m* =  − 1.3 mV) compared with repetition trials (*m* =  − 0.6 mV) (Gajewski et al., 2010); the amplitude of the N2 component per task differed significantly in repetition and switch conditions, *t*(21) = 2.75,* p* < .001, *d* = 0.56, *BF*_10_ = 4.23, and *t*(21) =  − 3.27, *p* < .004, *d* =  − 0.69, *BF*_10_ = 11.7, respectively. In contrast, the effect of the factor task did not reach significance, *F*(1, 21) < 0.01, *p* = .963, η_G_^2^ = 1 × 10^–6^.

The amplitude of the N2 component was further modulated by an interaction effect between the factors task and trial type, *F*(1, 21) = 25.34, *p* < .011, η_G_^2^ = .01, *BF*_10_ = 2168.87. In particular, we only obtained significant switch costs on the amplitude of the N2 component for the affective task, *t*(21) = 4.36, *p* < .001, *d* = 0.93, *BF*_10_ = 115.3, but not for the neutral task, *t*(21) =  − 0.73, *p* = .47, *d* =  − 0.16, *BF*_10_ = 0.28. More importantly, difference waves, calculated by subtracting repetition trial amplitudes from switch trial amplitudes for each task, indicated an increased switch-related negativity on the amplitude of the N2 component for the affective (*m* =  − 1.4 mV) compared with the neutral task (*m* = 0.2 mV), *t*(21) =  − 5.03, *p* < .001, *d* =  − 1.07, *BF*_10_ = 466.17 (Fig. 4B). Probably, these results reflect a less efficient task set implementation for the affective compared with the neutral task.

For the P3 component, we obtained the often-reported frontocentrally distributed negativity in switch relative to repetition trials, which was more visible in the affective compared with the neutral task condition (Fig. 4E and F). We detected a positive peak at Cz 514 ms after stimulus onset, which was used to specify the center of the analysis interval (± 75 ms). Within the interval, the amplitude of the P3 component was modulated by the factor trial type, resulting in a more positive amplitude in repetition (*m* = 7.8 mV) relative to switch trials (*m* = 7.2 mV), *F*(1, 21) = 4.88, *p* < .038, η_G_^2^ = .01 (Karayanidis et al., 2011); the amplitude of the P3 component per task differed for the repetition but not for the switch condition, *t*(21) = 5.06,* p* < .001, *d* = 1.08, *BF*_10_ = 490.9, and *t*(21) = 1.52, *p* = .144, *d* =  − 0.32, *BF*_10_ = 0.6, respectively. Furthermore, the effect of the factor task reached significance, *F*(1, 21) = 16.06, *p* < .001, η_G_^2^ = .03, which indicated a more positive amplitude in the affective (*m* = 8.1 mV) compared with the neutral task (*m* = 6.8 mV), which probably reflects an emotional expression effect (Elchlepp et al., 2021).

The amplitude of the P3 component was further modulated by the interaction effect between the factors task and trial type, *F*(1, 21) = 15.91, *p* < .001, η_G_^2^ = .01, *BF*_10_ = 111.53. In more detail, we only obtained significant switch costs on the amplitude of the P3 component for the affective task, *t*(21) = 3.43, *p* < .003, *d* = 0.73, *BF*_10_ = 16.14, but not for the neutral task, *t*(21) =  − 0.98, *p* = .34, *d* =  − 0.21, *BF*_10_ = 0.34. Difference waves for the switch-repetition contrast for each task indicated an increased switch-related negativity on the amplitude of the P3 component in the affective (*m* =  − 1.4 mV) compared with the neutral task (*m* = 0.2 mV), *t*(21) =  − 3.99, *p* < .001, *d* =  − 0.85, *BF*_10_ = 51.01 (Fig. 4B), which probably reflects a hampered task-set implementation in the affective compared with the neutral task (Zhou et al., 2024).

### Discussion

In Experiment 1, we measured the typical switch cost effect, with longer RTs in switch trials compared with repetition trials. More importantly, affective asymmetric switch costs emerged, reflected in larger switch costs when participants switched from the neutral towards the affective task compared with the reverse switch direction (Eckart et al., 2023; Reeck & Egner, 2015; Schuch et al., 2012).

Behavioral effects were mirrored at the neural level by a switch-related posterior positivity at Pz, which emerged within 400 ms and 700 ms after cue presentation. Most importantly, this switch-related posterior positivity was further modulated by affective task content, which indicates a neural correlate of affective asymmetric switch costs during the CTI. This effect was particularly evident in the difference waves, which showed an increased positive amplitude in the neutral compared with the affective task. These results are consistent with the assumption that the affective task content modulates ERPs of proactive control. These results imply a hampered anticipatory task-set reconfiguration when participants switch toward the affective task compared with switching toward the neutral task.

For the stimulus-locked ERP components, we obtained a frontocentral negativity in switch relative to repetition trials, which was further enlarged by the affective task content. This was especially evident in the difference waves, which showed an increased negativity on the amplitude of the N2 component and a decreased positivity on the amplitude of the P3 component for the affective compared with the neutral task. These results are consistent with previous evidence suggesting that the implementation of the task sets was less efficient in the affective compared with the neutral task (Zhou et al., 2024).

Together, the results of Experiment 1 revealed that the affective task content hampers proactive control by impairing the trial-by-trial initiation of the switching processes between tasks. Additionally, we replicated previous evidence that affective task content also modulated stimulus-related neural markers of the switching processes. As a follow-up question, we asked whether affective task content also modulates processes related to the maintenance of task-sets in working memory. These processes are related to the mixing costs (Braver et al., 2003). To address this, Experiment 2 examined whether affective task content modulates mixing costs, both at the level of the neural correlates and the behavioral markers.

## Experiment 2

In Experiment 2, we investigated whether affective task content modulates the neurocognitive dynamics of processes that are involved in controlling the maintenance of task sets while participants switch between different tasks. To that end, we focused on behavioral mixing costs and the mixing-related centroparietal positivity (Goffaux et al., 2006; Jost et al., 2008; Karayanidis & Jamadar, 2014; Karayanidis et al., 2011). According to the working memory account of mixing costs, these costs arise from the sustained demands of maintaining multiple task sets in an active state within working memory and from the selection of the appropriate task set on a trial-by-trial basis (Braver et al., 2003; Goffaux et al., 2006; Los, 1996; however, see Rubin & Meiran, 2005). According to many accounts, these processing demands contribute to the cognitive mechanisms, which enable the repeated and flexible change between different task sets (Braver et al., 2003; Kiesel et al., 2010; Wylie et al., 2009).

The mixing-related positivity, on the neural level, reflects an increased centroparietal positivity within 300 ms and 500 ms after cue onset, for repetition trials in mixing blocks compared with single-task trials in single-task blocks. This cue-locked centroparietal positivity presumably reflects a generic form of preparation required in repetition and switch trials but not in single-task trials, because it appears to index goal-setting as signaled by the cue (Goffaux et al., 2006; Karayanidis & Jamadar, 2014; Karayanidis et al., 2011; Steinhauser & Steinhauser, 2019; Steinhauser et al., 2021; Wylie et al., 2009). For Experiment 2, the joint measurement of the behavioral mixing costs and the mixing-related centroparietal positivity provided a means to further specify the neural and behavioral dynamics of cognitive control in affective task switching.

If affective task content modulates the maintenance of task-sets in working memory, then the mixing costs, i.e., the RT difference between repetition and single task trials, should differ between the affective and the neutral task, thus reflecting asymmetrical mixing costs. At the neural level, this should result in different amplitudes for the affective and neutral tasks in the cue-locked mixing-related centroparietal positivity between 300 and 500 ms at Pz (Karayanidis & Jamadar, 2014; Steinhauser & Steinhauser, 2019).

Alternatively, it is conceivable that the maintenance of the task-sets in working memory is unaffected by affective task content. This should result in a lacking difference for the RT mixing costs between the affective and the neutral task. In that case, we should not find different amplitudes for the affective and neutral tasks when analysing the cue-locked mixing-related centroparietal positivity between 300 and 500 ms at Pz.

Finally, for Experiment 2, we aimed to extend the findings from Experiment 1 by reducing the CTI from 1,200 ms to 700 ms. This allowed us to investigate whether the affective task content will modulate the cue-locked switch-related posterior positivity under a shorter preparatory interval. Additionally, we investigated whether affective task content also modulates stimulus-related neural markers of the switching process for a reduced preparatory interval.

### Methods

#### Participants

Twenty-four healthy right-handed participants (18 females and 1 diverse; mean (m) age = 23 years) were invited to take part in the experiment after obtaining written informed consent and were debriefed after the session. The procedure was identical to Experiment 1.

#### Task and procedure

The procedure was identical to Experiment 1, with the exception that the CTI was reduced from 1,200 ms to 700 ms and the mixing block number was reduced from 13 to 10 blocks to limit the duration of the experiment. As a result, participants performed 10 mixing blocks of 65 trials, resulting in 650 trials of task switching. Furthermore, we added two single-task blocks for the neutral and the affective tasks of 64 trials each, summing up to 128 trials of single-task trials per task. The single-task blocks were presented in randomized order before the start of the mixing blocks. After preprocessing, participants contributed to the affective repetition condition on average 129 trials (minimum = 118, maximum = 160). For the affective switch condition, on average, 127 trials (minimum = 115, maximum = 157). For the neutral repetition condition, on average, 131 trials (minimum = 125, maximum = 153). For the neutral switch condition, on average, 128 trials (minimum = 120, maximum = 154). For the single-task affective condition, participants contributed on average 95 trials (minimum = 62, maximum = 116), and for the single-task neutral condition, on average 103 trials (minimum = 70, maximum = 122). We always exclude participants for whom more than 40% of trials were rejected; one participant was excluded for this reason in the present experiment. Data acquisition was identical to Experiment 1.

### Data analysis

#### Behavioral data

The analysis of behavioral data was identical to Experiment 1. In more detail, for the RT analyses, trials with an erroneous response (*m* = 6.8%) and outliers that deviated more than ± 2.5 SD from the mean RTs for each participant and factor combination (*m* = 2.1*%*) were excluded from the dataset. For the calculation of mixing costs, we analyzed mean RTs and error rates separately with an ANOVA with the within-subject factors task (neutral, affective) and trial type (repetition trials, single-task trials) with a significance threshold of 5%. We applied paired two-sided Bonferroni-corrected *t*-tests to resolve relevant interaction effects. As for Experiment 1, we applied Bayesian statistics to further validate the obtained behavioral results.

### Electrophysiological data

The procedure was identical to Experiment 1, with the exception that all preprocessing steps were applied to the shortened CTI of 700 ms. For the stimulus-locked ERP components, the epoch length was extended by an additional 800 ms. Furthermore, we quantified the mixing-related centroparietal positivity at Pz within the CTI of 300 ms and 500 ms after cue onset (Steinhauser & Steinhauser, 2019). To analyze how affective task content modulates the mixing-related centroparietal positivity, we computed the mean amplitude of this ERP component based on the within-subject factors task (neutral, affective) and trial type (repetition trials, single-task trials). We utilized the mean amplitudes as a dependent variable in an ANOVA with a significance threshold of 5%. To further validate the ERP-related results, we applied Bayesian statistics, as in Experiment 1.

### Results

#### Behavioral data

##### Analysis of switch costs

We first tested for the effect of the factor task on RTs and obtained a significant main effect, *F*(1, 22) = 49.52, *p* < .001, η_G_^2^ = .13. Participants executed the neutral task (*m* = 517 ms) more rapidly compared with the affective task (*m* = 583 ms). In addition, we obtained a significant main effect of the factor trial type, *F*(1, 22) = 32.24, *p* < .001, η_G_^2^ = .13, with reduced RTs in the repetition condition (*m* = 520 ms) compared with the switch condition (*m* = 580 ms), which reflects switch costs on RTs (Wylie & Allport, 2000). In addition, the repetition and switch RTs per task showed significant differences, *t*(21) = 5.92, *p* < .001, *d* = 1.24, *BF*_*10*_ = 3563.70, and *t*(21) = 6.82, *p* < .001, *d* = 1.42, *BF*_*10*_ = 23,733.35, respectively.

**Fig. 5:**
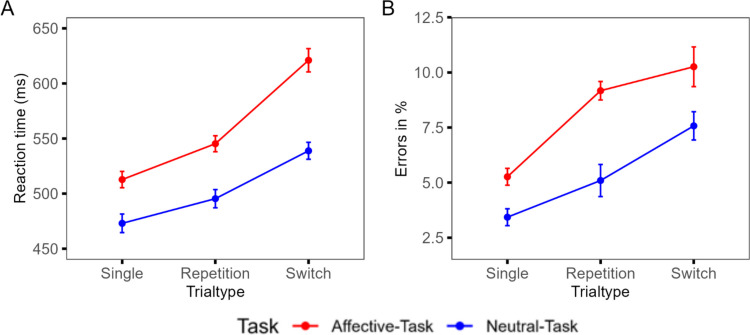
**A)** Mean reaction times (RTs) as a function of task and trial type. Depicted are single-task, repetition, and switch conditions. **B**) Error rates from single-task, repetition, and switch conditions. Error bars represent the standard error of the mean. The results indicate that the switch costs on RTs increase when participants switch from the neutral toward the affective task compared with the opposite switch direction. In contrast, the mixing costs for RTs were similar for both tasks, whereas the mixing costs in errors increased for the affective compared with the neutral task

More importantly, we obtained affective asymmetric switch costs on RTs as the interaction effect between the factors task and trial type reached significance, *F*(1, 22) = 13.6, *p* < .001, η_G_^2^ = .01, *BF*_*10*_ = 12. In detail, we obtained significant switch costs on RTs in both tasks, for the affective task, *t*(21) =  − 6.05, *p* < .001, *d* =  − 1.26, *BF*_*10*_ = 4639.73, and for the neutral task, *t*(21) =  − 4.27, *p* < .001, *d* =  − 0.89, *BF*_*10*_ = 100.46, respectively. Most importantly, the switch costs on RTs were increased in the affective task (*m* = 76 ms) compared with the neutral (*m* = 43 ms) task,* t*(22) =  − 3.63, *p* < .001, *d* =  − 0.76, *BF*_*10*_ = 25.01 (Fig. 5A). These results indicate that affective asymmetric switch costs emerge under the condition of a short preparatory interval, thus extending the findings observed with a longer preparatory interval as in Experiment 1.

Subsequently, we analyzed the error rates within the mixing blocks. We obtained a significant main effect of the factor trial type, *F*(1, 22) = 6.86, *p* < .016 η_G_^2^ = .03. Participants made more errors in switch trials (*m* = 9%) compared with repetition trials (*m* = 7.1%), which indicated switch costs in the error rates (Fig. 5B). The factor task reached significance, *F*(1, 22) = 15.55, *p* < .001, η_G_^2^ = .08, participants conducted more errors in the affective task (*m* = 9.7%) compared with the neutral task (*m* = 6.3%). The interaction effect between the factors task and trial type did not reach significance, *F*(1, 22) = 1.94, *p* = .177, η_G_^2^ = .00, *BF*_*10*_ = 0.65.

##### Analysis of mixing costs

Next, we compared RTs in single-task trials with that in repetition trials, for which we obtained a significant main effect of the factor trial type, *F*(1, 22) = 13.88, *p* < .001, η_G_^2^ = .04, which indicates mixing costs, i.e. faster responses in single-task trials (*m* = 493 ms) compared with repetition trials (*m* = 520 ms) (Fig. 5A) (Rubin & Meiran, 2005). We furthermore obtained a significant main effect of the factor task, *F*(1, 22) = 24.71, *p* < .001, η_G_^2^ = .09. Participants responded faster to the neutral task (*m* = 483 ms) compared with the affective task (*m* = 529 ms). The interaction effect between the factors task and trial type did not reach significance, *F*(1, 22) = 1.22, *p* = .281, η_G_^2^ = .00, *BF*_*10*_ = 0.42*.* A direct comparison of mixing costs on RTs for each task indicated similar costs, *t*(22) =  − 1.1, *p* = .281, *d* =  − 0.23, *BF*_*10*_ = 0.38, which is consistent with the assumption that the affective task content did not modulate mixing costs on RTs.

Furthermore, we compared the error rates from the single-task trials with those of repetition trials. We obtained a significant main effect of the factor trial type, *F*(1, 22) = 16.53, *p* < .001, η_G_^2^ = .01. Participants produced more errors in the repetition condition (*m* = 7.2%*)* compared with the single-task condition (*m* = 4.4%), which reflected mixing costs on the error rates. We furthermore obtained a significant main effect of the factor task, *F*(1, 22) = 36.12, *p* < .001, η_G_^2^ = .11. Participants produced more errors for the affective task (*m* = 7.3%) compared with the neutral task (*m* = 4.3%). Furthermore, the interaction effect between the factors trial type and task reached significance, *F*(1, 22) = 6.19, *p* < .021, η_G_^2^ = .017, *BF*_*10*_ = 4.35. The mixing costs were increased for the affective task (*m* = 3.9%) compared with the neutral task (*m* = 1.7%), which indicates that affective task content modulated the mixing costs on the error rates (Fig. 5B).

### Event-related brain potentials

#### Cue-locked event-related potentials

##### Switch-related potentials

**Fig. 6:**
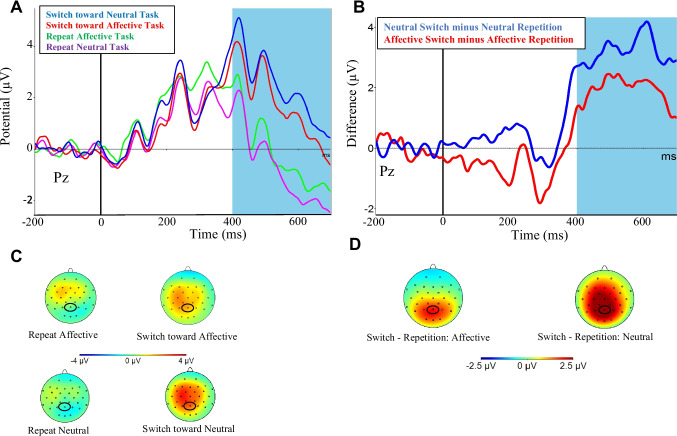
Cue-locked event-related potentials of correct trials at Pz during the cue-target interval, focusing on the switch-related posterior positivity. **A**) All four experimental conditions, whereas the blue area marks the time interval used for statistical testing based on Steinhauser and Steinhauser (2019). **B**) The difference waves of the neutral switch minus neutral repetition (blue) and the affective switch minus affective repetition (red) conditions. The topographical plot (**C**) is based on the four experimental conditions and (**D**) is based on the switch-repetition difference for the affective task and the neutral task, respectively. The topographical plots focus on the time interval used for statistical testing (i.e., 400 ms and 700 ms after cue onset). The difference wave for the neutral compared with the affective task showed an increased positivity, indicating a neural correlate of the affective asymmetric switch costs before stimulus onset, for a reduced preparatory interval compared with Experiment 1

Similar to Experiment 1, we observed a typical P3b-like positivity (Fig. 6A), which was further confirmed by the topographies showing a posterior spatial distribution (Fig. 6C and D). Mirroring the previously reported behavioral results, statistical analysis revealed a significant main effect of the factor trial type on amplitude, *F*(1, 22) = 65.39, *p* < .001, η_G_^2^ = .36. The amplitude of the posterior positivity was increased in the switch (*m* = 2.2 µV) compared with the repetition condition (*m* =  − 0.4 µV), which reflects a switch-related posterior positivity. Furthermore, the amplitude of the posterior positivity per task differed significantly for repetition and switch conditions, *t*(22) = 2.29, *p* < .032, *d* = 0.47, *BF*_*10*_ = 1.88, and *t*(22) =  − 2.46, *p* < .022, *d* =  − 0.52, *BF*_*10*_ = 2.52, respectively. Again, the factor task did not reach significance, *F*(1, 22) = .46, *p* = .506, η_G_^2^ = .00.

The amplitude of the switch-related posterior positivity was further modulated by affective task content, as indicated by the significant interaction effect between the factors task and trial type, *F*(1, 22) = 8.52, *p* < .008, η_G_^2^ = .03, *BF*_*10*_ = 32.54. In detail, we obtained significant switch costs on the amplitude of the posterior positivity for the affective task, *t*(22) =  − 4.39, *p* < .001, *d* =  − 0.92, *BF*_*10*_ = 130.89, and for the neutral task, *t*(22) =  − 9.98, *p* < .001, *d* =  − 2.1, *BF*_*10*_ = 8 × 10^6^, respectively. Most relevant, Fig. 6B depicts the difference waves (i.e., switch minus repetition amplitudes per task), which showed that the amplitude of the switch-related posterior positivity was increased in the neutral task (*m* = 3.2 µV) compared with the affective task (*m* = 1.9 µV), *t*(22) =  − 2.45, *p* < .008, *d* = 2.1, *BF*_*10*_ = 5.95. The results of Experiment 2 extend the previous findings on the neural correlates of the affective asymmetric switch costs for task-switching conditions with a reduced CTI.

##### Mixing-related potentials

**Fig. 7:**
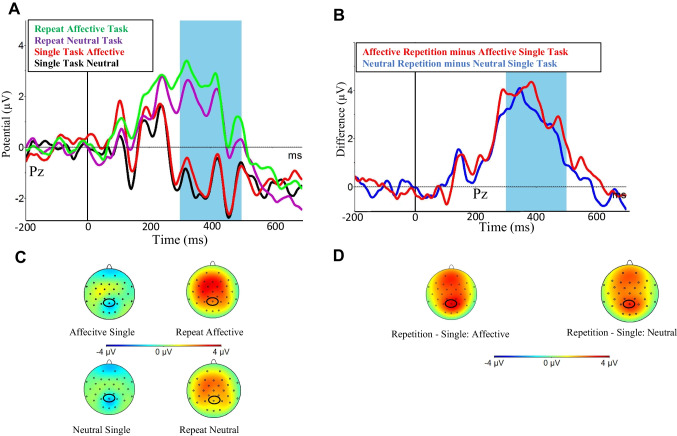
Cue-locked event-related potentials of correct trials at Pz during the cue-target interval, focusing on the mixing-related centroparietal positivity. **A**) All four experimental conditions, the blue area marks the time interval used for statistical testing based on Steinhauser and Steinhauser (2019). **B**) The difference waves of the neutral repetition minus neutral single task condition (blue) and the affective repetition minus affective single task (red) condition. The topographical plot (**C**) is based on the four experimental conditions and (**D**) is based on the switch-repetition difference for the affective task and the neutral task, respectively. Both topographical plots focus on the time interval used for statistical testing (i.e., 300 ms and 500 ms after cue onset). The difference waves for the neutral compared with the affective task were not different, indicating that affective task content was not modulating this neural correlate of proactive control

Next, we analyzed the cue-locked mixing-related potentials, for which we obtained an increased positivity over centroparietal sites in repetition compared with single-task trials (Fig. 7A) (Goffaux et al., 2006; Steinhauser & Steinhauser, 2019). The inspection of the topographies of the component indicates a more centroparietal spatial distribution compared with the cue-related ERP components (Fig. 7C and D). Behavioral results were mirrored on the neural level by a significant main effect of the factor trial type on amplitude, *F*(1, 22) = 42.29, *p* < .001, η_G_^2^ = .33. This resulted in an increased amplitude of the centroparietal positivity in repetition (*m* = 1.8 mV) compared with single-task trials (*m* =  − 1.4 mV). Furthermore, the factor task reached significance, *F*(1, 22) = 6.18, *p* < .021, η_G_^2^ = .01, which reflected a more positive amplitude in the affective (*m* = 0.4 mV) compared with the neutral task (*m* =  − 0.1).

The amplitude of the centroparietal mixing-related positivity was *not* further modulated by affective task content, as reflected by the nonsignificant interaction effect between the factors task and trial type, *F*(1, 22) = 1.97, *p* = .174, η_G_^2^ = .00, *BF*_*10*_ = 0.69. This is also illustrated by the similar difference waves for the neutral and the affective tasks, *t*(22) = 1.4, *p* = .174, *d* =  − 0.23, *BF*_*10*_ = 0.52, which suggests that the affective task content had not modulated this neural correlate of proactive control (Braver et al., 2003; Goffaux et al., 2006) (Fig. 7B).

#### Stimulus-locked event-related potentials

##### Switch-related potentials

**Fig. 8:**
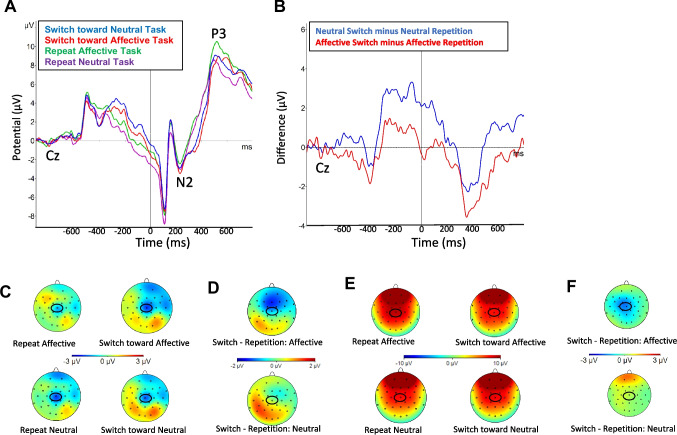
Stimulus-locked event-related potentials of correct trials at Cz, which focuses on the switch-related negativity. **A**) All four experimental conditions, marking the N2 and P3 components, respectively. **B**) The difference waves of the neutral switch minus neutral repetition condition (blue) and the affective switch minus affective repetition (red) condition, respectively. The topographical plots (**C**) and (**D**) depict the N2 component (i.e., 100-ms interval centered on the N2 peak), the former showing the four experimental conditions and the latter depicting the switch-repetition difference for the affective task and the neutral task, respectively. The topographical plots (**E**) and (**F**) focus on the P3 component (i.e., 150-ms interval centered on the P3 peak), the former showing the four experimental conditions and the latter depicting the switch-repetition difference for the affective task and the neutral task, respectively. The difference wave for the neutral compared with the affective task showed a decreased negativity in the N2 and an increased positivity in the P3 window, reflecting a neural correlate of the affective asymmetric switch costs after stimulus onset, for a reduced preparatory interval compared with Experiment 1

For the stimulus-locked ERP components, we obtained a frontocentrally distributed switch-related negativity, which was again more pronounced in the affective compared with the neutral task condition (Fig. 8A, C, and D) (Karayanidis & Jamadar, 2014). For the N2 component, we measured a negative peak at Cz 230 ms after stimulus presentation, which was used to define the center of the analysis interval (± 50 ms).

Within the interval, the amplitude of the N2 component was neither modulated by the factor task nor by the factor trial type, *F*(1, 22) = 1.18, *p* = .29, η_G_^2^ = .00, and *F*(1, 22) = 1.76, *p* = .198, η_G_^2^ = .00, respectively. In contrast, the interaction effect between the factors task and trial type reached significance, *F*(1, 22) = 5.65, *p* < .027, η_G_^2^ = .00, *BF*_10_ = 5.05. In detail, we only obtained significant switch costs on the amplitude of the N2 component for the affective task,* t*(22) = 2.24, *p* < .036, *d* = 0.5, *BF*_10_ = 1.73, but not for the neutral task, *t*(22) =  − 0.78, *p* = .443, *d* =  − 0.16, *BF*_10_ = 0.28. Furthermore, the amplitude of the N2 component per task differed significantly for the repetition but not for the switch condition, *t*(22) = 2.57,* p* < .017, *d* = 0.54, *BF*_10_ = 3.1, and *t*(22) =  − .66, *p* = .513, *d* =  − 0.14, *BF*_10_ = 0.27, respectively. More importantly, difference waves, calculated by subtracting repetition trial amplitudes from switch trial amplitudes for each task, indicated an increased switch-related negativity on the amplitude of the N2 component in the affective (*m* =  − 0.8 mV) compared with the neutral task (*m* = 0.2 mV), *t*(22) =  − 2.37, *p* < .027, *d* =  − 0.5, *BF*_10_ = 2.19 (Fig. 8B). These results are consistent with the assumption of a less efficient task-set implementation in the affective compared with the neutral task.

For the P3 component, we obtained a commonly reported frontocentrally distributed negativity in switch relative to repetition trials, which was, as in Experiment 1, more visible in the affective compared with the neutral task (Fig. 8A, E, and F). We detected a positive peak at Cz 522 ms after stimulus onset, which was used to specify the center of the analysis interval (± 75 ms).

Within this interval, the amplitude of the P3 component, showed a trend for being modulated by the factor task (1, 22) = 4.05, *p* = .057, η_G_^2^ = .00, whereas the factor trial type failed to reach significance, (1, 22) = 2.95, *p* = .100, η_G_^2^ = .00. In contrast, the amplitude of the P3 component was modulated by an interaction effect between the factors task and trial type, *F*(1, 22) = 21.01, *p* < .001, η_G_^2^ = .01, *BF*_10_ = 653.54. In detail, we only obtained switch costs on the amplitude of the P3 component for the affective task,* t*(22) = 3.94, *p* < .001, *d* =  − 0.8, *BF*_10_ = 48.54, but not for the neutral task, *t*(22) =  − 1.41, *p* = .173, *d* =  − 0.3, *BF*_10_ = 0.52. Furthermore, the amplitude of the P3 component per task differed significantly for the repetition condition but not for the switch condition, *t*(22) = 4.79,* p* < .001, *d* = 1.00, *BF*_10_ = 310.67, and *t*(22) =  − 0.67, *p* = .509, *d* =  − 0.14, *BF*_10_ = 0.29, respectively. More relevantly, difference waves for the switch-repetition contrast per task indicated an increased switch-related negativity on the amplitude of the P3 component in the affective (*m* =  − 1.5 mV) compared with the neutral task (*m* = 0.5 mV), *t*(22) =  − 4.48, *p* < .001, *d* =  − 0.9, *BF*_10_ = 195.59 (Fig. 8B). These results demonstrate a consistent modulation of the switch-related negativity by the affective task content across Experiments 1 and 2, which indicates that stimulus-related neural markers of the switching process are modulated by affective task content for different preparatory conditions.

### Discussion

For Experiment 2, we obtained symmetrical mixing costs, reflected by similarly increased RTs in repetition compared with single-task trials for both the affective and neutral tasks. Mirroring behavioral results, we measured a mixing-related centroparietal positivity, emerging after cue onset within 300 ms and 500 ms over centroparietal sites. This mixing-related centroparietal positivity remained unchanged by the affective task content. This absence of modulation was especially evident in the difference waves for the neutral and affective tasks, consistent with the assumption that affective task content did not modulate this neural correlate of proactive control.

Interestingly, asymmetrical mixing costs emerged in the error rates, reflecting increased mixing costs for the affective compared with the neutral task. This result suggests that control processes, such as maintaining task sets in working memory and selecting the relevant one, were less efficient when participants had to select the affective task. However, this effect was not observed in processing time, as mixing costs in RTs remained comparable between affective and neutral tasks. Taken together with the finding that the mixing-related centroparietal positivity was not modulated by affective task content, it is conceivable that affective task content interfered with task-related implementation processes upon stimulus presentation.

We could further demonstrate that affective asymmetric switch costs also emerged under conditions of a reduced preparatory interval, as compared with Experiment 1, which were accompanied by a modulation of the amplitude of the switch-related posterior positivity after cue onset between 400 and 700 ms. The results showed that the amplitude of the switch-related posterior positivity was more positive when participants switched toward the neutral task compared with the affective task. As a result, we could show that the affective task content modulates the neural correlates of proactive control even for situations in which the degree of preparation is reduced compared with Experiment 1.

As for Experiment 1, the affective task content modulated the switch-related negativity on the amplitudes of the N2 and P3 components. This was especially evident in the difference waves, for which we obtained a more negative switch-related negativity in the affective compared with the neutral task on the amplitudes of the N2 and P3 components, respectively. These results suggest a less efficient implementation of the affective compared with the neutral task set. Together, these results imply that proactive control is impeded by the affective task content at shorter and longer preparatory intervals. Additionally, we replicated results, which indicate that the affective task content also modulates stimulus-related neural markers of the switching process.

### General discussion

In this study, we investigated whether affective task content modulates the neural and behavioral markers of proactive control during affective task switching. We did so by investigating whether the cue-locked switch-related posterior positivity and the switch costs would be modulated by affective task content; in addition, we investigated whether the affective task content also modulates stimulus-related neural markers of the switching process. The two experiments featured a cued task-switching paradigm, in which participants were required to repeat and switch between neutral and affective tasks, with either a CTI of 1,200 ms or 700 ms. The results indicated a reduced amplitude of the switch-related posterior positivity in the affective compared with the neutral task. These neural results were mirrored on the behavioral level by affective asymmetric switch costs, with larger costs when participants switched towards the affective task compared with the neutral task (Eckart et al., 2023; Reeck & Egner, 2015). These results provide support for the assumption that the anticipatory task-set reconfiguration in the affective compared with the neutral task was less efficiently initiated across varying preparatory switching conditions.

As an additional question, we investigated whether the affective task content also modulates the cue-locked mixing-related centroparietal positivity, as well as behavioral mixing costs (Braver et al., 2003; Goffaux et al., 2006). In contrast to the modulation of the switch-related posterior positivity, the affective task content did not modulate the mixing-related centroparietal positivity, aligning with the symmetrical mixing costs on RTs. These results indicate that, especially the initiation of the switching process towards the affective task was hampered, but not the general goal-setting to prepare for either task.

Finally, in addition to the novel evidence that affective task content modulated proactive control processes, we replicated and extended previous findings that the affective task content also modulated stimulus-related neural markers of the switching process. This was reflected in a modulation of the stimulus-locked switch-related negativity, on the amplitude of the N2 and P3 components, for shorter and longer preparatory intervals (Zhou et al., 2024). Together, these results indicate that the affective task content interfered with the anticipatory reconfiguration of the affective compared with the neutral task set, as well as with the implementation of the affective task set relative to the neutral task set.

### Neural correlates of affective asymmetric switch costs

Across Experiments 1 and 2, we obtained evidence consistent with the assumption that the affective task content modulates cognitive control dynamics before stimulus onset. This interpretation is based on the reduction of the switch-related posterior positivity in the affective compared with the neutral task during the preparatory interval, accompanied by affective asymmetric switch costs. These findings provide support for the assumption of a less efficient anticipatory task-set reconfiguration in affective compared with neutral tasks, extending previous evidence from neurocognitive studies on affective task switching (Eckart et al., 2023; Reeck & Egner, 2015; Zhou et al., 2024).

These results relate to current theories of cognitive control, which consider task-set reconfiguration as a multicomponent process, which is reflected in both the cue-locked switch-related posterior positivity and the stimulus-locked switch-related negativity (Jamadar et al., 2010; Karayanidis & Jamadar, 2014; Rubinstein et al., 2001). According to this view, the cue-locked switch-related posterior positivity reflects proactive reconfiguration processes, such as goal-shifting, which updates the contents of working memory. The stimulus-locked switch-related negativity is related to stimulus-triggered processes that complete task-set reconfiguration upon stimulus presentation. Together, the current results suggest that the affective task content interferes with proactive *and* stimulus-related processes, as reflected by the modulation of the corresponding ERP components. These results indicate that the initiation and the implementation of the switching processes are impeded in affective compared with neutral tasks, likely contributing to the observed affective asymmetric switch costs.

A puzzling issue needs to be discussed, which relates to the question of whether the observed switch cost pattern on RTs might stem from inherent differences in task difficulty between the affective and neutral tasks. This is because the affective task showed longer RTs and increased error rates compared with the neutral task, which indicates that it was more difficult. Therefore, theoretically, it cannot be ruled out that differences in task difficulty may have affected the current result pattern in addition to or instead of the affective task content. For example, Kaiser et al. (2023) reported an fMRI study, which showed no differences in neural fMRI activity when the difficulty of performing affective and nonaffective cognitive control tasks was matched. While these findings might suggest that difficulty and not the affective task content has driven the effect pattern, we refrain from a difficulty explanation for the current data pattern as a sole account.

First, under the assumption that differences in task difficulty would have solely caused the effects on the RT switch costs and consequently on the amplitude of the related ERP components, we would expect increased switch costs for the neutral compared with the affective task and a lower amplitude of the switch-related posterior positivity, respectively. This should be the case, because switching between a difficult, and an easy task has often been shown to result in increased RT switch costs for the easy compared with the more difficult task (Eckart et al., 2023; Poljac & Yeung, 2014; Schuch et al., 2012; Wylie & Allport, 2000; Yeung & Monsell, 2003). However, in our case, an opposite switch cost pattern emerged, with larger switch costs for the affective, i.e., the more difficult task compared with the neutral task.

Furthermore, Kaiser et al. (2023) reported fMRI data as a parameter for the neural activation, which is known to provide increased neural activation in situations with increased RTs (Yarkoni et al., 2009). A lack of performance difference between two conditions might therefore require rather sophisticated data processing to reveal differences in neural fMRI activation (Kaiser et al., 2023). However, in the current study, we focused on ERP components, i.e., the switch-related posterior positivity, for which studies have shown that conditions with lower RTs (easy tasks) compared with higher RTs are often accompanied by a larger amplitude of the ERP component during the preparatory interval (Jost et al., 2008; Karayanidis et al., 2011; Lavric et al., 2008; Poljac & Yeung, 2014). Therefore, the current observation of higher RT switch costs and a lower amplitude of the switch-related posterior positivity when participants switched toward the affective compared with the neutral task is opposite to the result pattern, which would be expected if difficulty differences between tasks had solely caused the effect pattern.

In addition, we note that, in the literature, the result pattern regarding the influence of affective task content on the neural activity is heterogeneous across studies. For example, a recent meta-analysis of fMRI studies provided evidence consistent with the assumption that affective task content indeed modulates cognitive control in interference tasks and the related neural activity (i.e., emotional Stroop task) (Song et al., 2017). The authors reported consistently increased neural activation for affective compared with neutral task content in conflict trials in different regions of the prefrontal cortex related to cognitive control, such as the dorsolateral and medial prefrontal cortex. These effects were larger for intensive compared with milder affective interference conditions (Song et al., 2017), which complements the lack of difference in neural activity between affective and neutral tasks as observed by Kaiser et al. (2023).

To sum up, even though task difficulty, theoretically, may modulate task switching performance, it is unlikely that difficulty differences between tasks can explain the current switch cost pattern in the current affective task switching condition as a sole account. In any case, further investigation is required to elucidate how affective task content modulates cognitive control processes under different levels of task difficulty between neutral and affective tasks (Kaiser et al., 2023; Song et al., 2017).

We would like to elaborate on how different research traditions interpret processing-related differences between affective and neutral tasks. Related to the research field on cognitive control, Reeck and Egner (2015) proposed that affective compared with neutral tasks require increased inhibition, and this persisting inhibition increases the switch costs when participants switch towards the affective rather than switching towards the neutral task. This approach emphasizes the role of executive processes (i.e., inhibition) for the management of affective and neutral task sets to enable flexible behavior (Eckart et al., 2023).

Indeed, research on face processing provides evidence for the assumption of processing-related differences between affective and neutral tasks. This stems from EEG studies investigating face processing, which consistently report a protracted positive deflection with a frontocentral distribution as early as 120 ms until 300 to 400 ms poststimulus for emotional expressions (especially fearful expressions) compared with neutral expressions in face stimuli. This emotional expression effect also modulates other well-documented ERP components of face processing, such as the N170 (Eimer & Holmes, 2007; Hinojosa et al., 2015; Schindler et al., 2019). Only recently, it was reported that the emotional expression-related modulation of the N170 was absent in patients for whom the right amygdala had been removed compared with a control group with intact amygdala (Framorando et al., 2021). This suggests that the emotional expression effect on the N170 relies on the integrity of the amygdala, potentially to enable the generation of representations of emotional content to guide behavior. Future studies should aim to integrate evidence from these research areas by investigating whether switching towards an affective task also modulates the emotional expression effect on the N170 or whether this neural correlate of emotional processing remains invariant (for a similar approach, see Elchlepp et al., 2021).

### Affective task content modulates switching-related but not mixing-related neural correlates of proactive control

In the present study, we focused on how affective task content modulates neural correlates of proactive control. To this end, we applied mixed and single-task blocks, allowing us to analyze how affective task content influences the switching-related and mixing-related positivities—neural markers considered to reflect proactive control processes (Steinhauser & Steinhauser, 2019). These neural correlates of proactive control were complemented by the measurement of behavioral switch and mixing costs, which capture different processes of cognitive flexibility (Braver et al., 2003; Goffaux et al., 2006).

Our results indicate that affective task content interfered with the control processes required to initiate a switch between tasks. Specifically, the switch-related posterior positivity was reduced in the affective compared with the neutral task, accompanied by affective asymmetric switch costs. These neurocognitive findings indicate that proactive control processes, which operate on a trial-to-trial basis, were less efficient in initiating the switch towards the affective compared with the neutral task.

In contrast, affective task content did not modulate the neural correlate of general preparatory control, as indicated by the mixing-related centroparietal positivity. Previously, this centroparietal positivity was linked to a preparatory mechanism of goal-setting triggered by the cue (Karayanidis & Jamadar, 2014; Steinhauser & Steinhauser, 2019). The results indicate that this process of goal-setting was not modulated by affective task content, which was reflected by similar mixing-related positivities in the neutral and affective tasks and symmetrical mixing costs in RTs. These findings support the assumption that affective task content primarily interferes with the proactive reconfiguration of the task set before stimulus presentation but not with goal-setting itself.

The results of the present study provide further validation of ERP components to study cognitive control dynamics in task switching. Specifically, the switch-related posterior positivity and the mixing-related centroparietal positivity prove to be effective tools for examining affect-control processing asymmetries. Future research may build on this approach to explore how affective task content modulates further neurocognitive mechanisms.

### Conclusion

We provide novel evidence that affective task content modulates neural correlates of proactive control before stimulus onset. Indicated by a reduction of the switch-related posterior positivity in the affective compared with the neutral task, suggesting a less efficient anticipatory task-set reconfiguration. The mixing-related centroparietal positivity, on the other hand, was unaffected by affective task content, which suggests that general goal-setting was not impeded. This implies that the affective task content hampered the initiation of the switching processes between tasks. These results provide novel insights into how affective processing modulates the neural correlates of proactive control.

## Data Availability

The data generated and/or analyzed (or code used) during the current study are available from the corresponding author upon reasonable request.

## Footnotes

1 The authors thank Nim Tottenham for the possibility of using stimulus material from this data base. Please contact Nim Tottenham at nlt7@columbia.edu for more information concerning the stimulus set. Development of the MacBrain Face Stimulus Set was overseen by Nim Tottenham and supported by the John D. and Catherine T. MacArthur Foundation Research Network on Early Experience and Brain Development.

2 We thank an anonymous reviewer for this suggestion.
